# The production of excretory-secretory molecules from *Heligmosomoides polygyrus bakeri* fourth stage larvae varies between mixed and single sex cultures

**DOI:** 10.1186/s13071-021-04613-9

**Published:** 2021-02-08

**Authors:** Marta Maruszewska-Cheruiyot, Ludmiła Szewczak, Katarzyna Krawczak-Wójcik, Magdalena Głaczyńska, Katarzyna Donskow-Łysoniewska

**Affiliations:** Laboratory of Parasitology, General Karol Kaczkowski Military Institute of Hygiene and Epidemiology, Warsaw, Poland

**Keywords:** *Heligmosomoides polygyrus bakeri*, Nematode, Excretory-secretory products, Pheromones, Mass spectrometry

## Abstract

**Background:**

Excretory-secretory (ES) products are crucial in maintaining helminths in the host. Consequently, the proteins of ES are potential vaccine molecules and potential therapeutic agents for autoimmune diseases. *Heligmosomoides polygyrus bakeri*, a gastrointestinal parasite of mice, is a model of hookworm infection in humans. ES produced by both sexes of *H. polygyrus bakeri* L4 stage cultured separately shows different immunomodulatory properties than ES obtained when both sexes are cultured together. Accordingly, the objective of this study was to identify and compare the excretory-secretory molecules from single-sex and mixed cultures.

**Methods:**

The composition of ES of male and female L4 stage nematodes in the presence (cultured together) or absence (cultured alone) of the opposite sex was examined. Proteins were identified using mass spectrometry. The functions of identified proteins were explored with Blast2GO.

**Results:**

A total of 258 proteins derived from mixed larval culture in the presence of sex pheromones were identified, 160 proteins from pure female cultures and 172 from pure male cultures. Exposure of nematodes to the sex pheromones results in abundant production of proteins with immunomodulatory properties such as Val proteins, acetylcholinesterases, TGF-β mimic 9 and HpARI. Proteins found only in ES from mixed larval cultures were TGF-β mimics 6 and 7 as well as galectin.

**Conclusions:**

The presence of the opposite sex strongly influences the composition of ES products, probably by chemical (pheromone) communication between individuals. However, examination of the composition of ES from various conditions gives an opportunity for searching for new potentially therapeutic compounds and anthelminthics as well as components of vaccines. Manipulation of the nematode environment might be important for the studies on the immunomodulatory potential of nematodes.
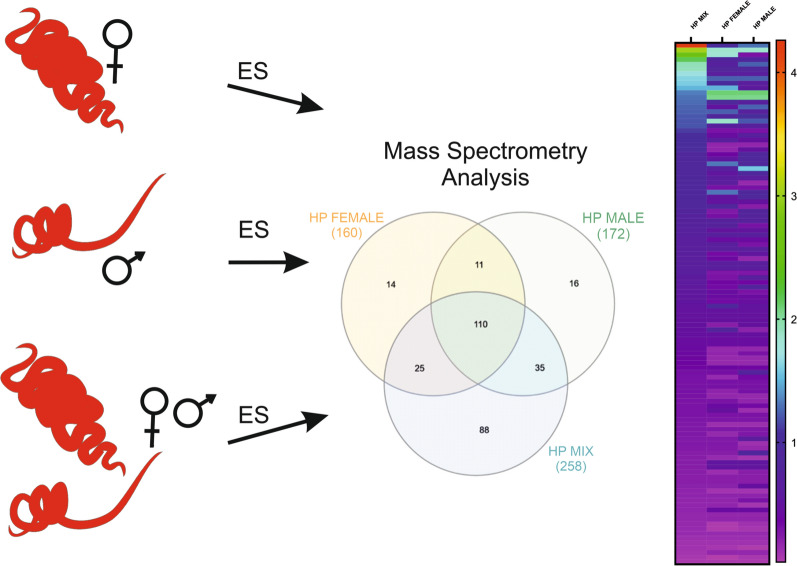

## Background

Helminths including nematodes influence the host to survive and raise the level of their reproduction. One of their important strategies is the production of excretory-secretory (ES) products, a set of various compounds that influence the host. The composition of parasite ES varies depending on the species; however, it frequently contains many molecules with various functions such as proteins, glycoproteins, glycans and glycolipids [1].

Some of the immunogenic proteins found in ES may be vaccine candidates [2]. Other ES-derived molecules could be effective drugs against autoimmune diseases and allergies [3]. ES-derived compounds that influence various populations of immune cells employ several signaling pathways [4, 5]. Among them are molecules specific for the parasite. However, some helminth proteins mimic host products such as macrophage migration inhibitory factor (MIF) or transforming growth factor β (TGF-β) [6–8]. These elements of ES modulate the immune response of the host. Many studies of parasitic nematode ES composition have been conducted using proteomic methods. Organisms studied include *Dirofilaria immitis, Ancylostoma caninum, Brugia malayi, Nippostrongylus brasiliensis, Strongyloides ratti, Teladorsagia circumcincta, Trichinella pseudospiralis, Trichinella spiralis, Toxocara canis, Ascaris suum* and *Haemonchus contortus* [9–18].

Most nematode species are dioecious. Some analyses of ES composition have shown differences between male and female-derived products [19–22]. In our previous studies, we observed that ES derived from male and female L4 stage of gastrointestinal parasite of mice, *Heligmosomoides polygyrus bakeri*, had different influences on immature JAWS II dendritic cells (DC). Moreover, JAWS II cells showed more immunomodulation when stimulated with mixed sex products [23]. Similarly, transfer of DC pre-exposed to live nematode L4 of *H. polygyrus bakeri* in vitro into mice with colitis induced with dextran sulfate sodium (DSS) showed that only DC pre-exposed to nematode females inhibited disease development [24]. However, when male and female parasites were used together for DC stimulation, the condition was exacerbated (unpublished data). Based on these results, our hypothesis is that sex pheromones produced by male and female nematodes influence the production of excretory-secretory molecules. To study this, we examined ES of *H. polygyrus bakeri* L4 stage males and females cultured in vitro alone (in the absence of opposite sex pheromones) and both together (in the presence of opposite sex pheromones). To investigate the ES composition, we used mass spectrometry and bioinformatic analysis.

## Methods

### Parasite preparation

The experiments were conducted on the BALB/c strain of mice. Eight-week-old pathogen-free males were allowed to adjust to laboratory conditions for 7 days before the start of the experiment at the animal house facilities in the General Karol Kaczkowski Military Institute of Hygiene and Epidemiology. Animals were placed in groups of five in cages with room temperature maintained at 24–25°C, humidity 50%, under a 14/10-hour light/ dark cycle and allowed *ad libitum* access to drink and commercial pellet food. The mice were orally infected with 300 infective larvae (L3) of *H. polygyrus bakeri* and then killed 6 days after infection when the parasite was in the fourth stage. The larvae were prepared as described by Maruszewska-Cheruiyot et al. [23]. Briefly, the larvae were collected and the sex of the L4 stage determined by locating the presence of the bursa at the caudal end of pre-male larvae. The larvae were washed five times with medium and then immediately used for ES preparation. Two independent experiments were conducted on ES obtained from nematode culture collected from independently infected mice.

### ES obtainment

Nematodes were cultured at a density of 600 larvae per ml in RPMI 1640 medium (Biowest) supplemented with L-glutamine 2 mM (Biowest), penicillin (100 U/ml) and streptomycin (100 μg/ml), without fetal bovine serum (FBS), at 37 °C. The first 24 h collection of ES was discarded; medium was supplied and harvested for the next 72 h. The supernatant was collected, centrifuged at 8,000×*g* for 10 min and concentrated with a 3-kDa filter (VWR); then it was sterilized through 0.2-µm filters. The final protein concentration of samples was measured by RC DC™ Protein Assay (Bio-Rad) and stored at − 80°C until analysis.

### Sample preparation

The mass spectrometry analysis was performed at the Mass Spectrometry Laboratory IBB PAN.

Two independent analyses of the ES samples were conducted. Protein solutions were subjected to the standard procedure of trypsin digestion, during which proteins were reduced with 0.5 M (5 mM) TCEP for 1 h at 60 °C, blocked with 200 mM MMTS (10mM) for 10 min at room temperature and digested overnight with 10 µl 0.1 µg/µl trypsin. The resulting peptide mixtures were applied in equal volumes of 20 µl to the RP-18 pre-column (Waters, Milford, MA, USA) using water containing 0.1% formic acid (FA) as a mobile phase and then transferred to a nano-HPLC RP-18 column (internal diameter 75 µM, Waters, Milford, MA, USA) using the ACN gradient (0–35% ACN in 160 min) in the presence of 0.1% FA at a flow rate of 250 nl/min. The column outlet was coupled directly to the ion source of the Orbitrap Elite mass spectrometer (Thermo Electron Corp., San Jose, CA, USA) working in the regime of data-dependent MS to MS/MS switch. A blank run ensuring the absence of cross-contamination from previous samples preceded each analysis.

### Analysis of mass spectrometry data and protein identification

The acquired MS/MS data were preprocessed with Mascot Distiller software (v. 2.6 or 2.7, MatrixScience, London, UK), and a search was performed with the Mascot Search Engine (MatrixScience, London, UK, Mascot Server 2.5) against the Nematoda proteins (1,283,514 sequences) deposited in NCBInr database (20190409, 198,058,131 sequences; 72,054,367,693 residues). To reduce mass errors, the peptide and fragment mass tolerance settings were established separately for individual LC-MS/MS runs after a measured mass recalibration. Methylothiolation (C) was set as a fixed modification and oxidation (M) as a variable modification. Peptide mass tolerance and fragment mass tolerance were set as ± 5 ppm and ± 0.01 Da, respectively. The rest of the search parameters were as follows: enzyme: trypsin; missed cleavages: 1; instrument: HCD. The Decoy option was activated for further target/decoy-based FDR control, and the peptide score threshold was adjusted to keep the FDR < 1%. The significance threshold was set at *p *< 0.01. The results were filtered using the Mascot Percolator. Proteins with score values of at least 70 were analyzed. One representative of two analyses of ES is presented.

### Bioinformatic analysis

Protein functional annotation was prepared using Omicsbox v1.3 software (Biobam). The initial Blast search was performed against the NCBI non-redundant (nr) database, with default parameters. Gene Ontology (GO) annotations were made with default parameters. GO annotation results were merged with the InterPro database scanning results and EggNOG v5.0 orthology and functional annotation data. A Venn diagram was prepared using InteractiVenn (https://doi.org/10.1186/s12859-015-0611-3).

## Results

### Quantitative differences between *H. polygyrus bakeri* L4 stage mix, female and male ES

**Fig. 1.**
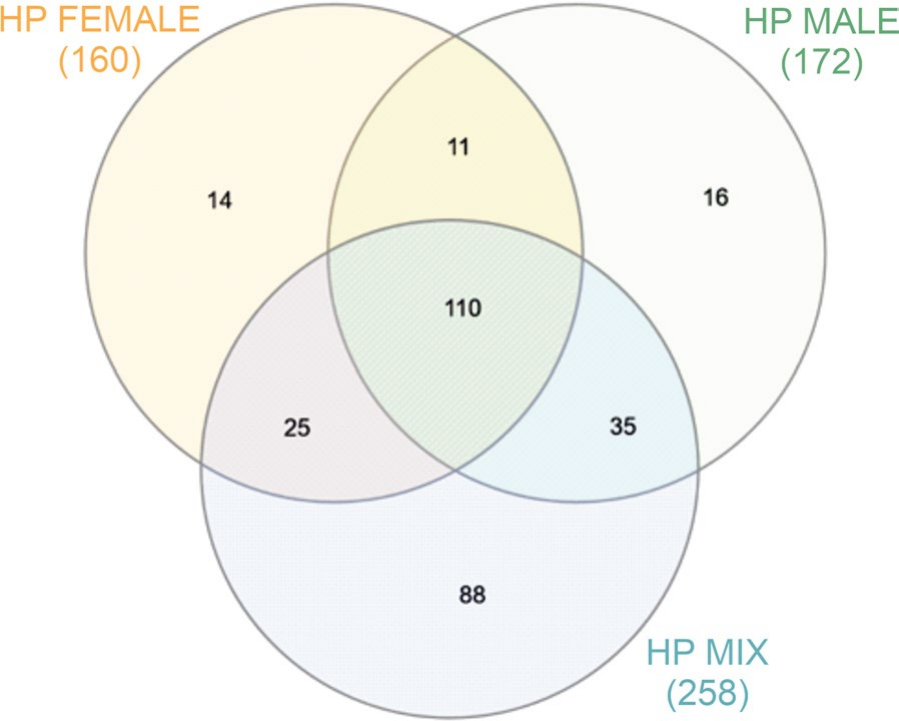
Venn diagram showing the distribution of proteins identified in excretory-secretory antigen (ES) of *H. polygyrus bakeri* L4 stage females (Hp female), males (Hp male) and a mix of both (Hp mix)

Analysis was performed on sets of proteins derived from Mascot identification results, having score values of at least 70. This included 258 proteins derived from mixed L4 ES, 160 from female ES and 172 from male ES. Most of the proteins were found in all groups (Fig. 1). In *H. polygyrus* male ES, 68% (117) proteins had GO annotations, and in *H. polygyrus* mixed ES and *H. polygyrus* female ES, 71% of proteins were annotated: 183 and 113 proteins, respectively.

### GO annotation of *H. polygyrus bakeri* L4 stage ES proteins

**Fig. 2.**
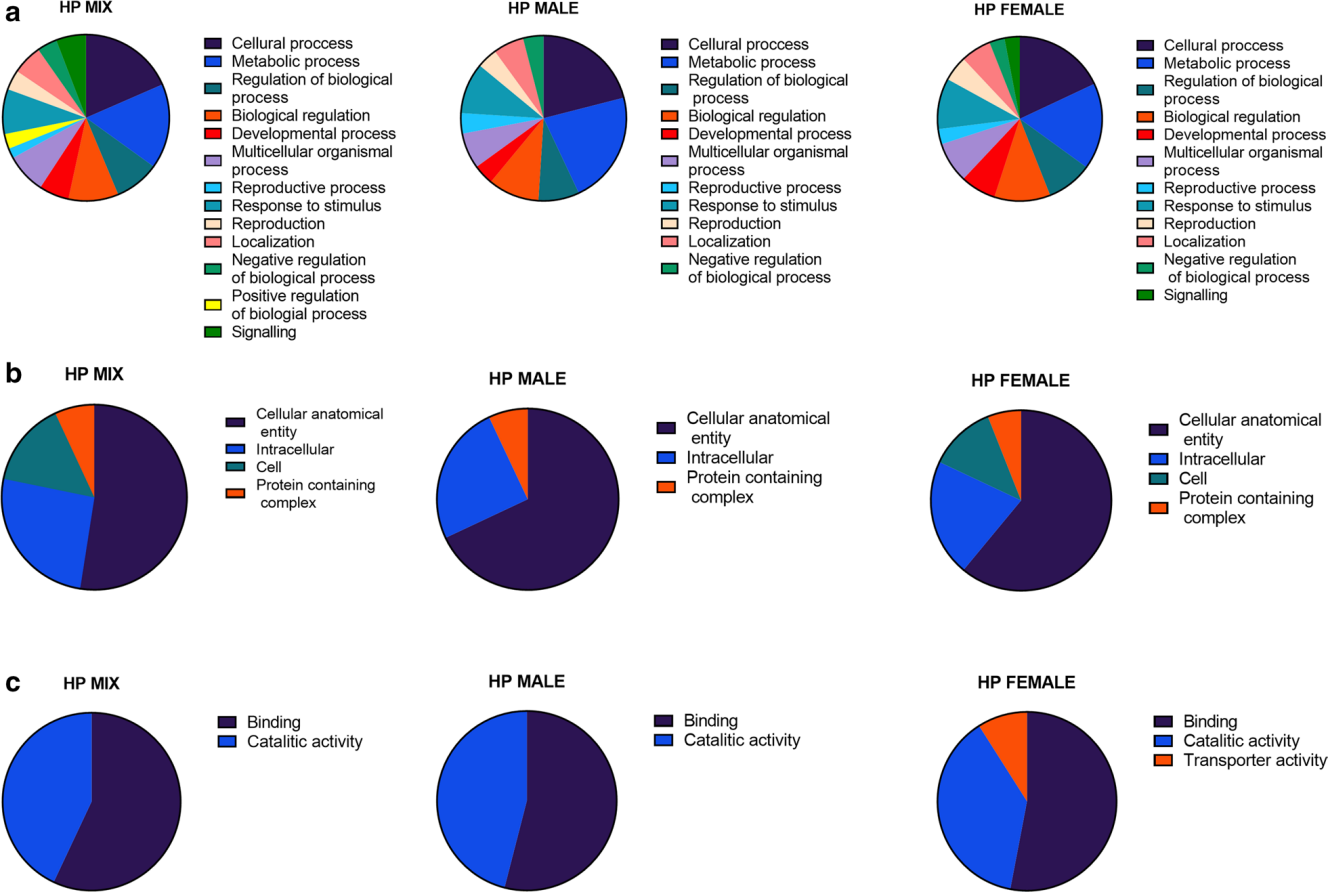
Comparison of Gene Ontology (GO) annotations on level 2 for excretory-secretory proteins of male, female or both together of *H. polygyrus bakeri* L4 stage. Identified proteins were analyzed with the OmicsBox program and based on the assigned biological process (**a**), cellular component (**b**) and molecular function (**c**). *H. polygyrus bakeri* L4 stage females (Hp female), males (Hp male) and a mix of both (Hp mix)

The most represented GO terms related to the biological process at level 2 were “cellular process” GO:0009987, “metabolic process” GO:0008152, “biological regulation” GO:0065007, “response to stimulus” GO:0050896 and “regulation of biological process” GO:0050789. In the mixed *H. polygyrus bakeri* and female *H. polygyrus bakeri* groups, there was the highest percentage of proteins with the “cellular process” term: 19% and 18%, respectively. In male *H. polygyrus bakeri* ES, there was a dominance of proteins with the “metabolic process” term (22%) and also a higher percentage of proteins related to the “cellular process” (21%) than in the other groups. In the mixed *H. polygyrus bakeri* group, there were unique proteins related to the GO term “positive regulation of biological process” GO:0048518. In turn, male *H. polygyrus bakeri* ES was the only group lacking proteins with the term “signaling” GO:0023052 (Fig. 2a).

Two of the most abundant terms related to molecular function at level 2 were “binding” GO:0005488—with the highest representation in the mixed sex group: 57% of annotated proteins—and “catalytic activity” GO:0003824—with the highest representation in the male *H. polygyrus bakeri* group: 46% of annotated proteins. Moreover, there was a unique term in the female *H. polygyrus bakeri* group–“transporter activity” GO:0005215–9% (Fig. 2b).

Within the cellular component category, most of the proteins were connected to the term “cellular anatomical entity” GO:0110165, with the highest percentage in male *H. polygyrus bakeri* (68%) and lowest in mixed *H. polygyrus bakeri*: 53%. The second most abundant term was “intracellular” GO:0005622 with the lowest percentage in female *H. polygyrus bakeri* (21%) and similar percentages in mixed *H. polygyrus bakeri* and male *H. polygyrus bakeri* (26% and 25%, respectively) (Fig. 2c).

At level 3, in the biological process category, there was a domination of proteins with terms “organic substance metabolic process” GO:0071704, “primary metabolic process” GO:0044238 and “nitrogen compound process” GO:0006807. In the molecular function category, the most abundant terms for all *H. polygyrus bakeri* ES groups were “ion binding” GO:0043167, “organic cyclic compound binding” GO:0097159, “heterocyclic compound binding” GO:1901363, “protein binding” GO:0005515, “small molecule binding” GO:0036094, “hydrolase activity” GO:0016787 and “catalytic activity, acting on protein” GO:0140096, where the two last terms were more represented in the male *H. polygyrus bakeri* group. In the cellular component category, there was the highest percentage of proteins related to “cytoplasm” GO:0005737, “membrane” GO:0016020 and “extracellular region” GO:0005576. The terms intracellular organelle” GO:0043229 and “organelle” GO:0043226 were highly represented in mixed *H. polygyrus bakeri*, whereas the term “extracellular region” GO:0005576 was more highly represented in *H. polygyrus bakeri* female and male groups than in the mixed *H. polygyrus* (Additional file 1: Figure S1).

### Differences in protein abundance between the ES produced by groups

Many proteins with immunomodulatory properties including a set of venom allergen/*Ancylostoma*-secreted protein-like (VAL) proteins, acetylcholinesterases, TGF-β mimic 9 and *H. polygyrus* alarmin release inhibitor (HpARI) were present in ES of all three examined groups. The majority of those proteins were most abundant in *H. polygyrus bakeri* mix ES.

**Fig. 3.**
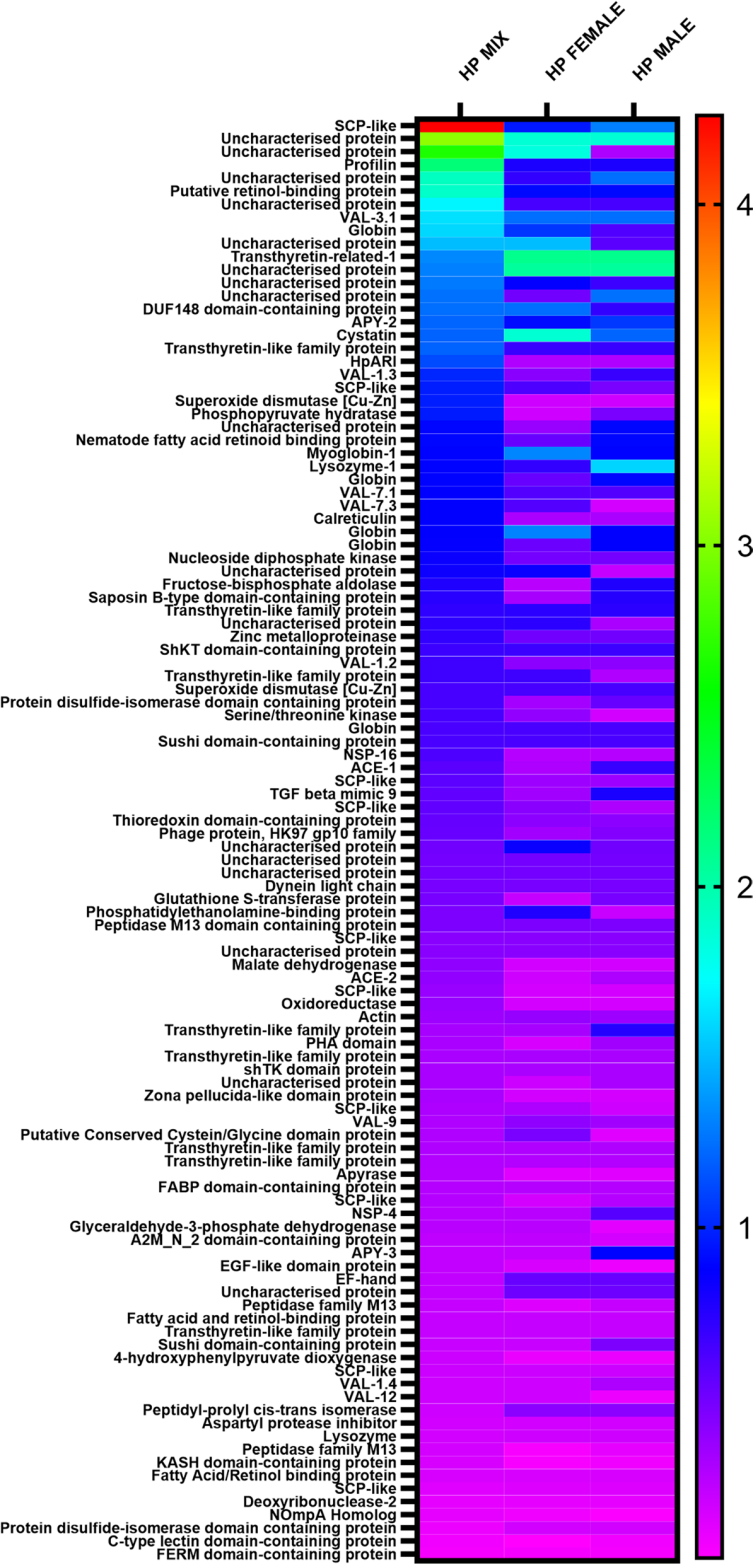
Comparison of protein abundance (emPAI) in ES products of *H. polygyrus* L4 stage females, males and a mix of both; *VAL* Venom allergen/Ancylostoma-secreted protein-like, *ACE* acetylcholinesterase, *NSP* novel secreted protein, *APY* apyrase, *HpARI* *H. polygyrus* alarmin release inhibitor. *H. polygyrus bakeri* L4 stage females (Hp female), males (Hp male) and a mix of both (Hp mix)

The most abundant *H. polygyrus bakeri* proteins from the mixed sex culture were SCP-like protein, profilin, VAL 3 isoform 1, putative retinol-binding protein, globins and a set of uncharacterized proteins. In both male and female ES one of the most abundant proteins was transthyretin-related protein 1. Moreover, cystatin and myoglobin-1 were the most abundant in female ES and lysozyme-1 in male ES (Fig. 3).

The set of proteins unique to female *H. polygyrus* ES included VAL-5 and transthyretin-like family proteins. Among unique male *H. polygyrus* ES proteins, there were VAL-6, apyrase, Sushi-domain containing protein and transthyretin-like family protein. The group of unique proteins from mixed *H. polygyrus* ES was very abundant and contained the TGF-β mimics 6 and 7, galectin, α-macroglobulin complement component, apyrases, serpin, VAL 7.2, 14, 17 and the Sushi-domain protein (Additional file 2: Table S2).

### COG categories of distribution in ES of *H. polygyrus bakeri* L4

**Fig. 4.**
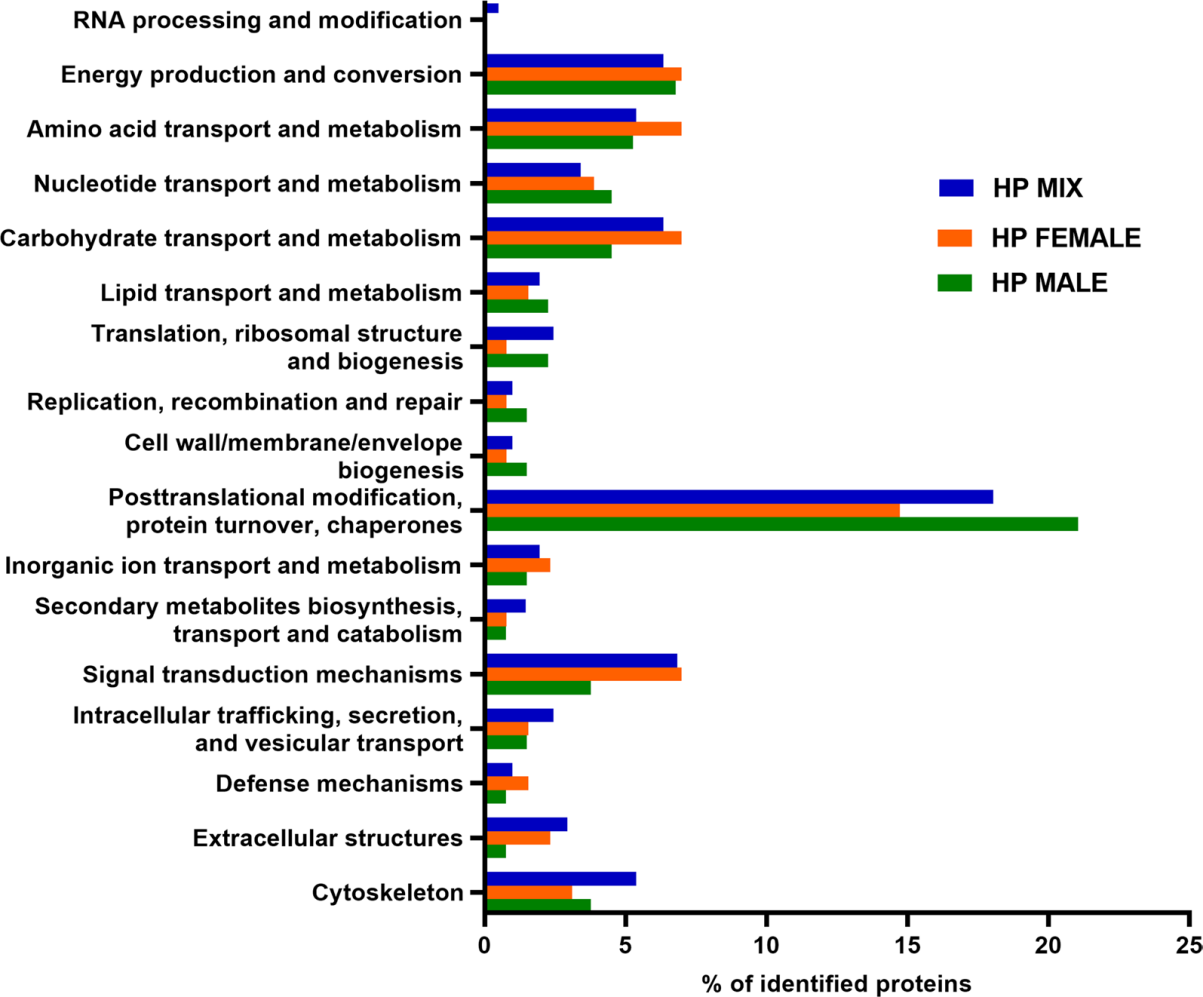
COG category distribution for ES proteins of *H. polygyrus bakeri* L4 stage females (Hp female), males (Hp male) and a mix of both, cultured both together (Hp mix)

In the EggNOG annotation results, there was a relatively high percentage of proteins in the COG category S-function unknown: 32% for mixed *H. polygyrus bakeri* ES and 38% for female *H. polygyrus bakeri* and male *H. polygyrus bakeri* ES (data not shown). Other abundant categories in COG were O: posttranslational modification, protein turnover, chaperones, with the lowest abundance in the Hp female group; C: energy production and conversion; E: amino acid transport and metabolism–with similar percentages of proteins in all groups and two categories with the lowest abundance in the *H. polygyrus bakeri* male group; G: carbohydrate transport and metabolism: T: signal transduction mechanisms (Fig. 4).

## Discussion

To our knowledge, this is the first study of the influence of nematode sex on the composition of excretory-secretory products. We used the intestinal mouse parasite, *H. polygyrus bakeri*, which is a model of mammal nematode infection [25]. The model is commonly used in various types of investigations such as the search for antiparasitic drugs, evolutionary aspects, host immune response against helminths, host-parasite interactions and use of the parasite as a source of compounds with potential therapeutic applications [26–29]. The latest studies show promising immunomodulatory abilities of *H. polygyrus bakeri* L4. The treatment effect of *H. polygyrus bakeri* infection against autoimmune disorders was observed before larval maturation [30–33]. Adults and L4 stage *H. polygyrus bakeri* ES protein composition were examined previously [34, 35].

We compared the proteins produced by L4 nematodes cultured in the presence or absence of the opposite sex. We used MS/MS analysis for three separate samples and described the biological process, cellular component and molecular function of the identified proteins. The composition of excretory-secretory products from female and male worms cultured together was different from that obtained from separate culture of males and females. The differences could be a result of communication between individual parasites. Information transfer between individuals of the same species is possible because of pheromones. Signal reception results in precise behavioral or physiological reactions [36]. Even immature *H. polygyrus bakeri* nematodes react in the presence of the opposite sex, moving toward target worms [37]. Communication in a single sex system based on pheromones is also observed. Female-to-female collaboration can have the function of bringing many females together to produce sufficient pheromones to attract males. However, *H. polygyrus bakeri* male-to-male attraction is weak [38]. Depending on the signal obtained, nematodes produce a different set of proteins. Go annotation of studied mixtures of ES support these findings. In ES from only males, there is a lack of proteins involved in signaling and positive regulation of the biological process with many involved in metabolic and cellular processes, confirming decreased male communication activity in this group.

Various immunomodulatory agents such as Val proteins, acetylcholinesterases, TGF-β mimic 9 and HpARI were most common in the ES obtained from nematodes harvested in the presence of the pheromones of the opposite sex, which suggests that communication between males and females increases the production of immunomodulators. The most abundant protein in ES from mixed nematodes was sperm coating-like protein (SCP like), also known as SCP/Tpx-1/Ag5/PR-1/SC7 (SCP/TAPS) family members, which have been observed in various eukaryote organisms, including nematodes. The best characterized groups of SCP-like proteins among parasitic worms are activation-associated proteins (ASPs) identified in *Ancylostoma caninum* and in related strongylid species [39]. The ASPs are abundant in *A. caninum* ES products and demonstrate immunomodulatory properties [40]. In addition, the most abundant proteins observed in ES of *H. polygyrus bakeri* were venom allergen-like proteins (VALs), also members of SCP/TAPS and homologues of ASPs [41]. SCP/TAPS proteins are also potential elements of vaccines against parasites [40].

The most abundant protein in ES of males and females not exposed to the opposite sex was transthyretin-related-1 protein. Transthyretins, transthyretin-like or transthyretin-related proteins have been identified in ES of other nematode species such as *Haemonchus contortus*, *Brugia malayi* and *Ascaris suum* [9, 12, 17]. One of the postulated functions of transthyretins proteins in nematodes is transfer of hormones [42, 43].

In addition, the group of exclusive proteins for ES collected from worms exposed to the opposite sex included TGF-β mimics 6 and 7 as well as galectin. Superfamily TGF-β proteins take part in the development of organisms [44]. TGF-β mimics are able to bind with the TGF-β receptors and activate signaling of the host. As a result, host regulatory T cells are activated; hence, TGF-β mimics are an important element of the immunomodulatory activity of *H. polygyrus bakeri* [45].

Galectins are multifunctional glycan-binding proteins with a role in the immune response [46]. The function of nematode galectins is not yet fully known; however, they are proposed to be critical for interactions with the host [47]. Galectins were identified as an element of ES of various nematode species, among them *B. malayi*, *D. immitis, A. caninum and T. circumcincta* [10, 13, 19, 48].

Chemical communication in *H. polygyrus bakeri* probably affects ES composition and consequently influences dendritic cells differently [23]. Multicellular parasites produce immunosuppressive molecules, and these can be used to treat autoimmune diseases [49]. However, a deeper investigation of pheromones and immunomodulatory abilities should be conducted to confirm the hypothesis.

Excretory-secretory molecules have been used in experimental studies of allergies, autoimmune and metabolic diseases [3]. Excretory-secretory nematode products show therapeutic effects in animal models of inflammatory bowel diseases (IBD) [50, 51], multiple sclerosis (MS) [52] and rheumatoid arthritis [53]. Some individual nematode proteins from excretory-secretory antigen have already been tested with a promising effect on experimental models of colitis and MS [54–56]. Examination of the composition of excretory-secretory products may reveal new therapeutic proteins as well as further opportunities to develop new anthelminthic drugs. However, manipulation of conditions of the nematode environment can influence the ES composition. Furthermore, infection with a single individual, typical of large nematodes, can influence their immunomodulatory effect.

## Conclusions

This study has shown that the composition of ES depends on whether it is derived from single sex or mixed nematode infections. The differences in ES composition may influence the effectiveness of modulating the immune response. The presence of the opposite sex strongly influences the composition of ES products, probably by chemical (pheromone) communication between individuals. However, examination of the composition of ES from various conditions gives an opportunity for searching for new potentially therapeutic compounds and anthelminthics as well as components of vaccines. Manipulation of the nematode environment might be important for the studies on the immunomodulatory potential of nematodes.

## Key findings

1. The production of excretory-secretory molecules is influenced by the presence of the opposite sex.
2. The presence of the opposite sex is associated with abundant production of immunomodulatory proteins.
3. TGF-β mimics 6, 7 and galectin are only found in excretory-secretory products from the mixed L4 stage of *H. polygyrus bakeri*.

## Supplementary Information

**Additional file 1: Figure S1. **Comparison of Gene Ontology (GO) annotations on level 3 for excretory-secretory proteins of male, female or both together of *H. polygyrus bakeri* L4 stage. Identified proteins were analyzed with the OmicsBox program and based on the assigned biological process (**a**), cellular component (**b**) and molecular function (**c**). *H. polygyrus bakeri* L4 stage females (Hp female), males (Hp male) and a mix of both (Hp mix).

**Additional file 2: Table S2.** Characteristics of proteins unique for ES of *H. polygyrus bakeri* females, males and mix of both.

## Data Availability

Data supporting the conclusions of this article are included within the article. The mass spectrometry proteomics data have been deposited to the ProteomeXchange Consortium via the PRIDE partner repository with the dataset identifier PXD023759 and 10.6019/PXD023759.

## Abbreviations

DC: Dendritic cells
DSS: Dextran sulfate sodium
ES: Excretory-secretory
FA: Formic acid
HpARI: *H. polygyrus* Alarmin release inhibitor
IBD: Inflammatory bowel diseases
MIF: Macrophage migration inhibitory factor
MS: Multiple sclerosis
SCP like: Sperm coating-like protein
TGF-β: Transforming growth factor β
VAL: Venom allergen/Ancylostoma-secreted protein-like
