# Supplementary figures and images for: The production of excretory-secretory molecules from Heligmosomoides polygyrus bakeri fourth stage larvae varies between mixed and single sex cultures
Source: Parasit Vectors. 2021 Feb 8;14:106. doi: 10.1186/s13071-021-04613-9 (PMC7871589; doi:10.1186/s13071-021-04613-9)

| Hp mix | Hp female | Hp male |
| --- | --- | --- |
| 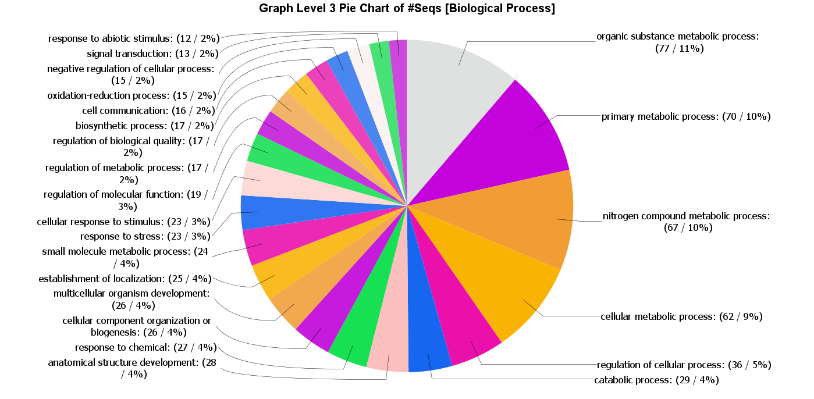 | 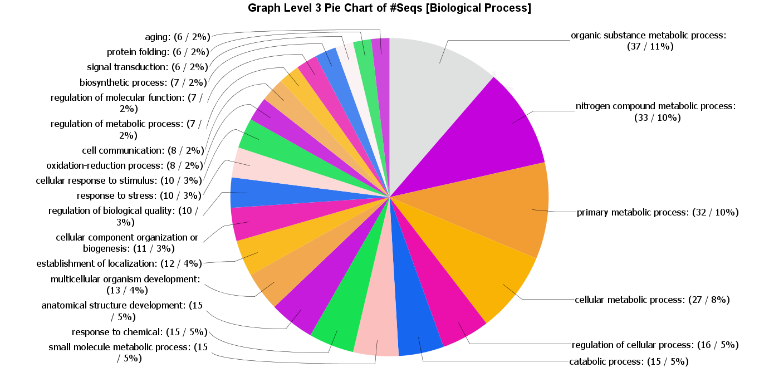 | 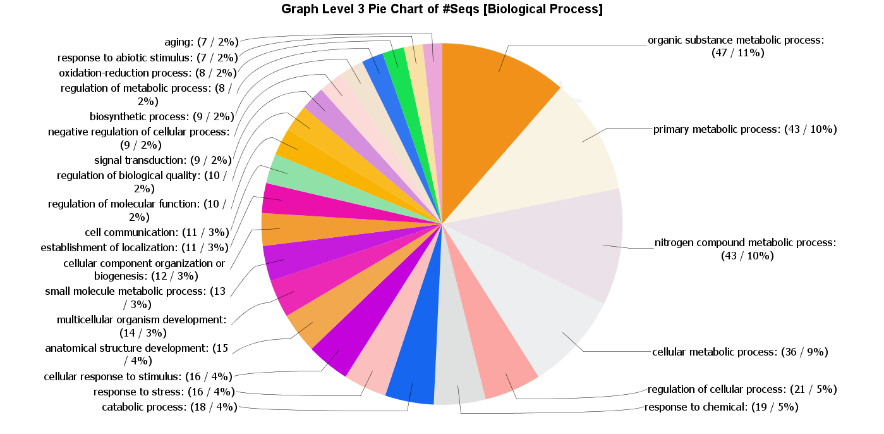 |
| 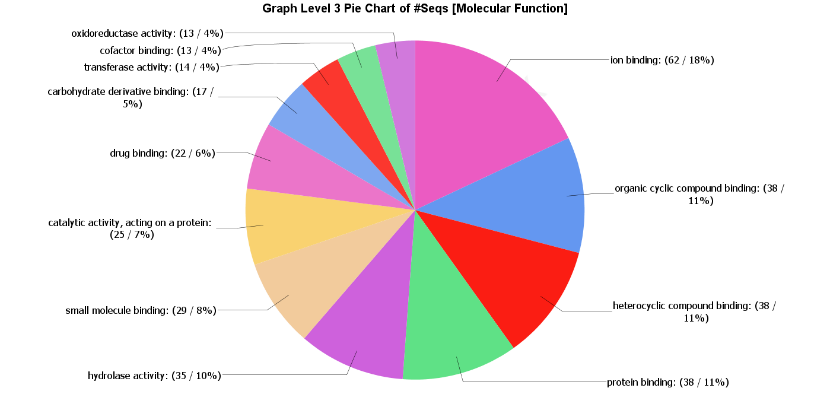 | 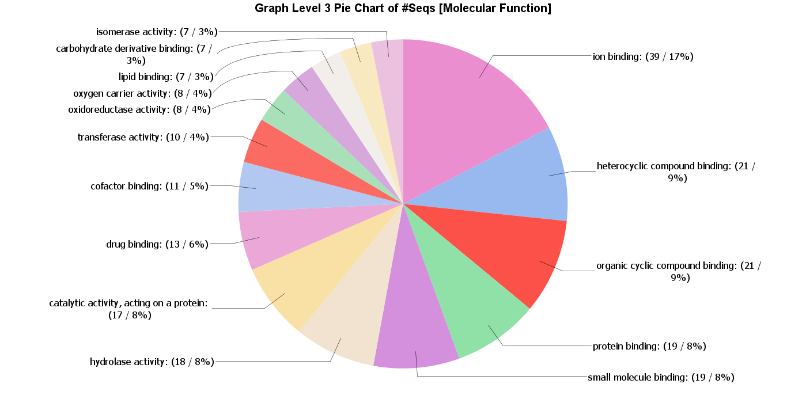 | 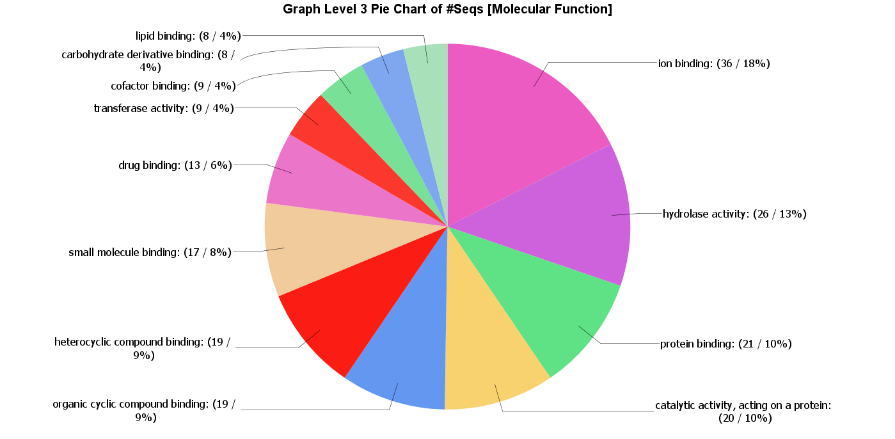 |
| 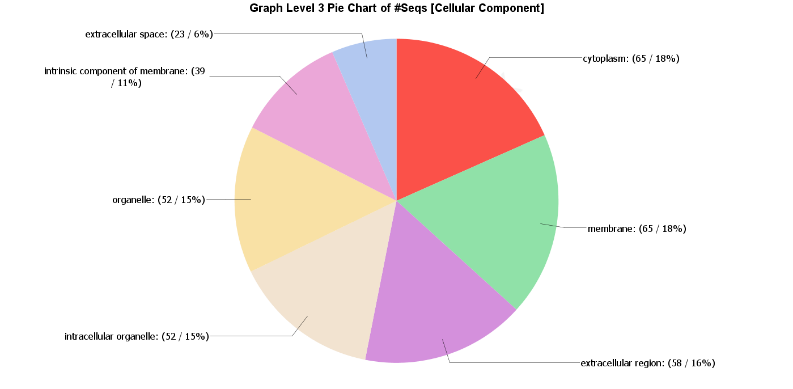 | 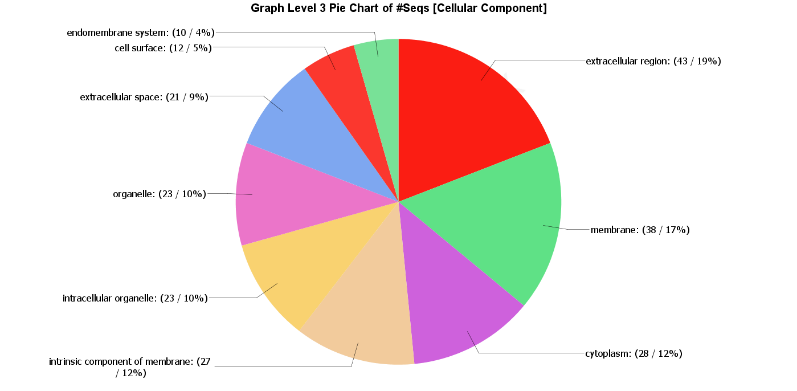 | 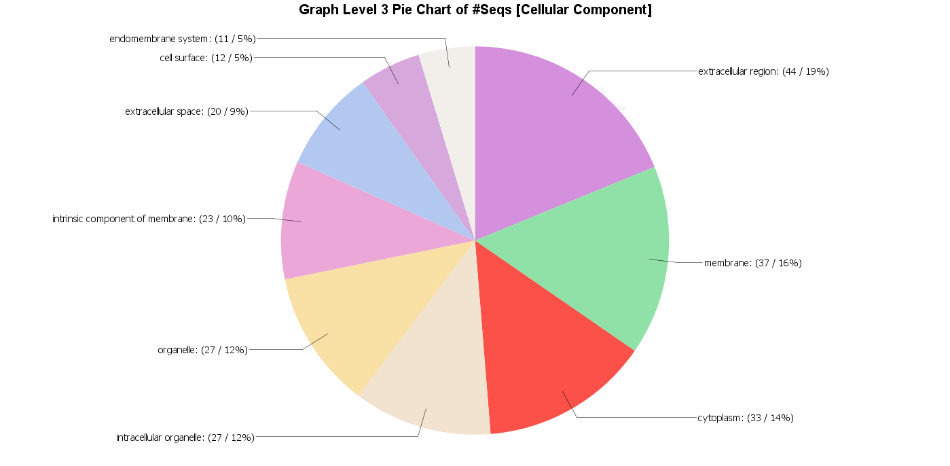 |

Supplement: Supplementary file 1 — Additional file 1: Figure S1. Comparison of Gene Ontology (GO) annotations on level 3 for excretory-secretory proteins of male, female or both together of H. polygyrus bakeri L4 stage. Identified proteins were analyzed with the OmicsBox program and based on the assigned biological process (a), cellular component (b) and molecular function (c). H. polygyrus bakeri L4 stage females (Hp female), males (Hp male) and a mix of both (Hp mix). [file 13071_2021_4613_MOESM1_ESM.docx]
